# Validating Physiological and Biomechanical Parameters during Intermittent Swimming at Speed Corresponding to Lactate Concentration of 4 mmol·L^−1^

**DOI:** 10.3390/sports8020023

**Published:** 2020-02-18

**Authors:** Gavriil G. Arsoniadis, Ioannis S. Nikitakis, Petros G. Botonis, Ioannis Malliaros, Argyris G. Toubekis

**Affiliations:** 1 Division of Aquatic Sports, School of Physical Education and Sports Science, National and Kapodistrian University of Athens, Dafne, 17237 Athens, Greece; garsoniadis@phed.uoa.gr (G.G.A.); inikitak@phed.uoa.gr (I.S.N.); pboton@phed.uoa.gr (P.G.B.); gmalliaros@phed.uoa.gr (I.M.); 2 Sports Performance Laboratory, School of Physical Education and Sport Science, National and Kapodistrian University of Athens, Dafne, 17237 Athens, Greece

**Keywords:** intermittent swimming, swimming training, arm stroke rate, arm stroke length, validity

## Abstract

Background: Physiological and biomechanical parameters obtained during testing need validation in a training setting. The purpose of this study was to compare parameters calculated by a 5 × 200-m test with those measured during an intermittent swimming training set performed at constant speed corresponding to blood lactate concentration of 4 mmol∙L^−1^ (V4). Methods: Twelve competitive swimmers performed a 5 × 200-m progressively increasing speed front crawl test. Blood lactate concentration (BL) was measured after each 200 m and V4 was calculated by interpolation. Heart rate (HR), rating of perceived exertion (RPE), stroke rate (SR) and stroke length (SL) were determined during each 200 m. Subsequently, BL, HR, SR and SL corresponding to V4 were calculated. A week later, swimmers performed a 5 × 400-m training set at constant speed corresponding to V4 and BL-5×400, HR-5×400, RPE-5×400, SR-5×400, SL-5×400 were measured. Results: BL-5×400 and RPE-5×400 were similar (*p* > 0.05), while HR-5×400 and SR-5×400 were increased and SL-5×400 was decreased compared to values calculated by the 5 × 200-m test (*p* < 0.05). Conclusion: An intermittent progressively increasing speed swimming test provides physiological information with large interindividual variability. It seems that swimmers adjust their biomechanical parameters to maintain constant speed in an aerobic endurance training set of 5 × 400-m at intensity corresponding to 4 mmol∙L^−1^.

## 1. Introduction

Swimming performance during training or a year-round training plan is dependent on several interrelated changes of physiological and biomechanical parameters [1,2]. Coaches aim to optimize swimmer’s training, facilitating performance improvement through the assessment of aerobic endurance and biomechanical parameters. Assessment of aerobic endurance parameters, such as speed corresponding to blood lactate concentration of 4 mmol∙L^−1^ (V4) or speed corresponding to lactate threshold and biomechanical parameters, requires testing with progressively increasing swimming speed protocols [3,4].

V4 has been highlighted as one of the most commonly used indices for assessing swimming endurance [5,6]. It is suggested that V4 corresponds to lactate threshold or the onset of blood lactate accumulation and may be used for the adjustment of training pace during training for improvement of aerobic capacity [4,7,8]. Additionally, biomechanical parameters corresponding to V4 may be connected to performance changes [9]. Calculation of V4 requires drawing of a speed vs. blood lactate concentration curve after the completion of several repetitions of 200-m to 400-m swimming with progressively increasing speed [10,11]. However, various progressively increasing speed tests have been used since 1980s. Specifically, progressively increasing speed tests conducted with five repetitions of 4-min, 6-min or 8-min duration in running [5,6], four to five repetitions of 200-m [8,9], seven repetitions of 200-m [3,10] or seven repetitions of 300-m and 400-m front crawl swimming [9] have been used for testing.

Despite the various progressively increasing swimming speed tests used, a criticism of the validity of using a fixed lactate concentration of 4 mmol∙L^−1^ as the concentration corresponding to lactate threshold has been raised [12] because of the high individual variability noticed on lactate concentration corresponding to the lactate threshold (i.e., 2–4 mmol·L^−1^ [13,14]). Whatever the case, testing the validity of predicting lactate concentration during a training set may be helpful for coaches since lactate concentration during endurance training sets may range from 2–6 mmol∙L^–1^ [12]. Furthermore, it is expected that physiological and biomechanical variables calculated by a progressively increasing speed test will be reproduced in a training set applied a few days later. However, calculation of V4 and all subsequent physiological (i.e., heart rate, lactate) and biomechanical parameters (i.e., stroke rate and stroke length) rely on mathematical calculations and linear or nonlinear correlations which all present a measurement error [4]. In this case, the parameters calculated by the progressively increasing speed test may differ from those measured during a long-duration training set, and this has never been examined.

To our knowledge, there is no study comparing fixed lactate values predicted by the speed vs. lactate curve with those obtained during an intermittent constant speed training set in well-trained swimmers. The purpose of the present study was to examine the validity of physiological and biomechanical parameters during intermittent swimming at speed corresponding to blood lactate concentration of 4 mmol∙L^−1^.

## 2. Materials and Methods

### 2.1. Participants

Twelve regional- and national-level competitive male swimmers specializing in various competitive distances volunteered to participate in the study (see Table 1). Participants had competitive swimming training background of mean (standard deviation: SD) 8.5 (1.7) years, and they participated in a daily swimming training (6 days per week) with duration of approximately 2 h per session. Each participant provided written informed consent after a thorough explanation of the study. The local institutional review board approved the experimental procedures (Approval No. 1007/26-4-2017), which were in accordance with the Declaration of Helsinki for Human Subjects.

### 2.2. Study Design

Physiological and biomechanical parameters calculated by a 5 × 200-m progressively increasing speed swimming test were compared with those measured during a constant speed 5 × 400-m intermittent swimming training set in this study. The speed during the training set was prescribed based on the speed vs. lactate concentration curve drawn after a progressively increasing speed swimming test.

### 2.3. Preliminary Testing—The 400-m Test

**Figure 1:**
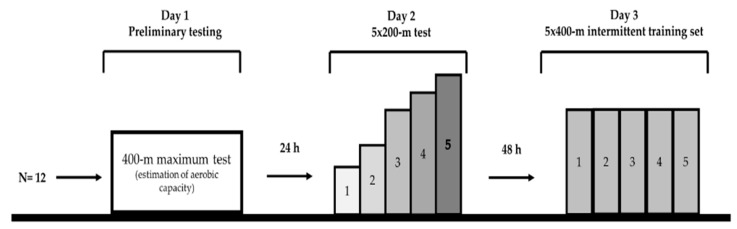
Study design of the current study; 400-m: 400-m front crawl; 5 × 200-m: five repetitions of 200-m front crawl, 5 × 400-m: five repetitions of 400-m front crawl, h: hours.

The study was conducted during the specific preparation mesocycle of training and swimmers were tested in three testing sessions 48 h apart (Figure 1). All swimming tests were conducted in a 25-m indoor swimming pool with a constant temperature of 25–26 °C. During the first visit and following a standardized warm-up (400-m slow swimming at 60% intensity, 4 × 50-m front crawl leg kicking, 4 × 50-m front crawl drills and 4 × 50-m front crawl swim with progressively increasing speed), swimmers participated in a 400-m front crawl test with maximum intensity. Immediately after the completion of the 400-m test, a face mask was applied to the swimmer for expired gas collection during recovery and VO_2_peak determination (VO2OOO; MedGraphics, Saint Paul, MN, USA; [15]).

### 2.4. Applying the 5 × 200-m Test

On the following day, all swimmers participated in a standardized swimming warm-up (following the same procedure as before the 400-m test) and 10 minutes later performed a 5 × 200-m front crawl test at intensities corresponding to 60%, 70%, 80% and 90% of the 200-m maximum speed progressively during the first four repetitions, exerting maximum effort in the last 200-m repetition. During the 5 × 200-m test, each repetition started every 5.5 min with a push-off start from within the water. Fingertip blood samples were collected after each repetition and were analyzed for blood lactate concentration (BL) using the reflectance photometry enzymatic reaction method (Accutrend Plus; Roche, Germany). Rating of perceived exertion (RPE) was indicated using a 10-point scale after each 200-m repetition [16]. Heart rate (HR) was recorded continuously using telemetry (s610i; Polar Electro, Oy, Kempele, Finland). V4 was determined for each swimmer by interpolation from a second-order polynomial function of swimming speed vs. lactate concentration data (mean (SD) R^2^ = 0.97 (0.03), mean r = 0.98 (0.01)). Heart rate corresponding to V4 (HR-V4) was determined for each swimmer individually by the linear regression between swimming speed and HR obtained during the 5 × 200-m test (mean R^2^ = 0.96 (0.05), mean r = 0.98 (0.02)). Stroke rate (SR) was calculated by the time (T) to complete three arm-stroke cycles (180·T^−1^), and stroke length (SL) was calculated by dividing swimming speed every 50 m (V) by SR. SR and SL corresponding to V4 (SR-V4 and SL-V4) were calculated by the interpolation of the best-fit regression line of SR and SL vs. swimming speed during the 5 × 200-m test (SR: mean R^2^ = 0.97 (0.03), mean r = 0.99 (0.01); SL: mean R^2^ = 0.98 (0.02), mean r = 0.99 (0.01)). Similarly, the rating of perceived exertion (RPE) corresponding to V4 was calculated by interpolation (mean R^2^ = 0.97 (0.02), mean r = 0.98 (0.01)).

### 2.5. Intermittent Swimming Training Set of 5 × 400-m

Swimmers completed an intermittent swimming training set 48 h after the completion of the 5 × 200-m test. A standardized warm-up as described for the previous testing sessions was applied before the training set. Ten minutes after warm-up, swimmers completed a 5 × 400-m training set at a constant speed corresponding to V4 with a resting interval of 30 to 45 s between repetitions to allow blood sampling. Swimming speed was kept constant by using a sound transmitter attached next to the swimmer’s ear (FINIS tempo pro, Finis Inc., Livermore, CA, USA) and according to the individual V4 that was determined by the 5 × 200-m test. Swimmers were advised to touch the swimming pool wall with their legs in each 25-m lap when hearing the transmitted sound. Additionally, one of the researchers recorded the time for each 50-m split in all 5 × 400-m repetitions (HS-80; CASIO, Guangzhou, China), and the mean speed of the test was calculated (V-5×400). BL concentration was collected after the first, third and fifth 400-m repetitions, while HR was recorded continuously and RPE was indicated after each repetition. SR and SL were calculated during each 400-m repetition of the 5 × 400-m swimming training set. The mean values of, BL-5×400, HR-5×400, RPE-5×400, SR-5×400 and SL-5×400 were used for the statistical analysis.

### 2.6. Statistical Analysis

Student’s *t*-test for paired samples was used to compare physiological and biomechanical parameters corresponding to V4 and calculated after the 5 × 200-m test with those measured during the 5 × 400-m intermittent swimming training set with constant speed. Specifically, V4, BL-V4, HR-V4, RPE-V4, SR-V4 and SL-V4 were compared to V-5×400, BL-5×400, HR-5×400, RPE-5×400, SR-5×400 and SL-5×400. Pearson *r* correlations were used to examine the relationship between relevant parameters. Additionally, the effect size for paired comparisons was calculated with Cohen’s *d* [17], using the pooled standard deviation as the denominator. The effect size was considered small if the absolute value of Cohen’s *d* was less than 0.20, medium if it was between 0.20 and 0.50 and large if it was greater than 0.50. The 95% confidence limits (95% CL) were also calculated for the mean differences between parameters obtained by the two tests. Agreement of measured parameters was tested using Bland and Altman plots [18]. SPSS, software (v.23, SPSS Inc., Chicago, IL, USA) was used for data analysis. Data are presented as mean and SD (in parentheses). Statistical significance was set at *p* < 0.05.

## 3. Results

### 3.1. Swimming Speed Comparison between Tests

Swimming speed prescribed by the 5 × 200-m test (V4) was successfully reproduced during the 5 × 400-m training set (V4 = 1.325 (0.08) m·s^−1^; V-5×400-m = 1.287 (0.10) m∙s^−1^; mean difference (SD): 0.04 (0.08) m·s^−1^; 95% CL: 0.060, 0.017 m·s^–1^; *d* = −0.42, *p* = 0.122). Additionally, significant correlation was noticed between V4 and V-5×400-m (r = 0.64, *p* = 0.02) and agreement was indicated by Bland and Altman plot (bias (SD): −0.04 (0.08) m∙s^−1^).

### 3.2. Comparison of Physiological Variables and Rating of Perceived Exertion between Tests

**Figure 2:**
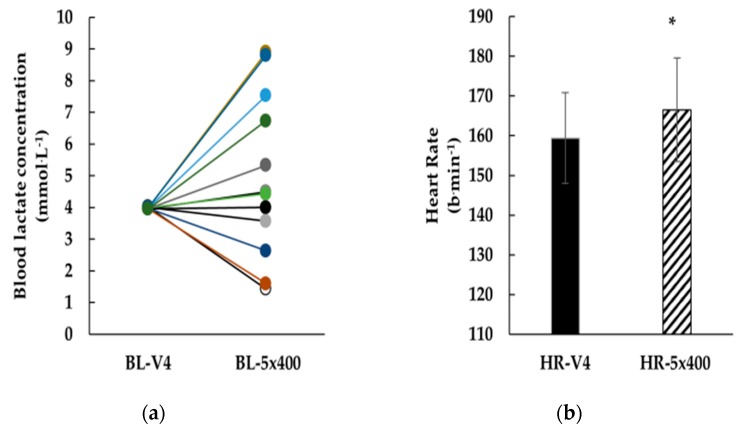
(**a**): Comparison between blood lactate concentration corresponding to V4 calculated after a progressively increasing speed 5 × 200-m test (BL-V4) and blood lactate measured during a constant speed intermittent swimming training set of 5 × 400-m (BL-5×400). (**b**): Comparison between heart rate corresponding to V4 calculated after a progressively increasing speed 5 × 200-m test (HR-V4) and heart rate measured during a constant speed intermittent swimming training set of 5 × 400-m (HR-5×400). *: p < 0.05 between HR-V4 and HR-5×400-m.

Following the training set, BL-5×400 was not different compared to 4 mmol·L^−1^ (BL-V4 = 4.0 (0.0) mmol·L^−1^; BL-5×400 = 5.0 (2.6) mmol·L^−1^; mean difference (SD): −1.0 (2.6) mmol·L^−1^; 95% CL: −1.53, −0.42 mmol·L^−1^; *d* = 0.75, *p* = 0.231, Figure 2). Bland and Altman plot indicated agreement between calculated and measured values (bias (SD): −1.0 (2.6) mmol·L^−1^). Training HR-5×400 was higher compared to HR-V4 (mean difference (SD): −7.2 (10.7) b·min^−1^; 95% CL: −11.22, −3.11 b·min^−1^; *d* = 0.59, *p* = 0.04, Figure 2) and these parameters were correlated (r = 0.63, *p* = 0.03). Bland and Altman plot showed agreement between calculated and measured values (bias (SD): 7 (11) b·min^−1^).

The calculated RPE values were successfully reproduced during the training set (RPE-V4 = 4.25 (2.09); RPE-5×400 = 5.17 (1.69); mean difference (SD): −1.0 (2.5); 95% CL: −1.6, −0.4; *d* = 0.52, *p* = 0.224]. Calculated and measured values were not correlated (r = 0.16, *p* = 0.60). Bland and Altman plot showed agreement between calculated and measured values (bias (SD): −1.0 (2.5)).

### 3.3. Comparison of Biomechanical Variables between Tests

**Figure 3:**
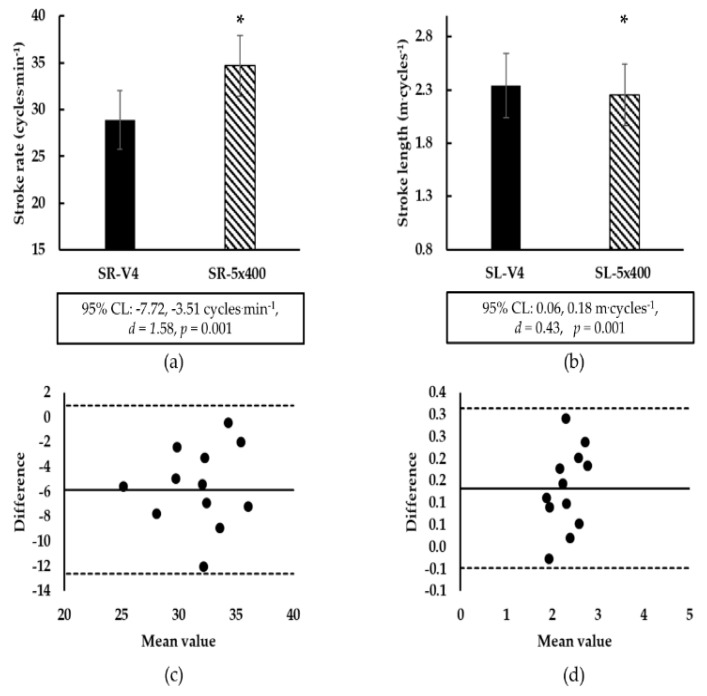
(**a**): Comparison of the stroke rate. (**b**): comparison of stroke length. Values corresponding to V4 and calculated after a progressively increasing speed 5 × 200-m test (SR-V4 and SL-V4) are compared with the stroke rate and stroke length measured during an intermittent constant swimming speed 5 × 400-m training set (SR-5×400 and SL-5×400). The 95% confidence limits (95% CL) and effect size (*d*) are indicated under the figures. (**c**): Bland and Altman plots of mean vs. difference in SR-V4 and SR-5×400 and (**d**): Bland and Altman plots of mean vs. difference in SL-V4 and SL-5×400. V4: speed corresponding to a blood lactate concentration of 4 mmol·L^−1^. Units of measure in (c) and (d) are not shown for clarity and are the same as in the corresponding figure (**a**) and (**b**) panel. * p < 0.05 between SR-V4 and SR-5×400 and between SL-V4 and SL-5×400-m.

Measured SR during the training set was increased compared to calculated SR and measured SL was decreased compared to calculated SL (Figure 3). Although a significant correlation was noticed between measured and calculated SR and SL values (SR, r = 0.57, *p* = 0.05; SL, r = 0.96, *p* = 0.001). Bland and Altman plot indicated agreement between calculated and measured for SR and SL (bias (SD): −5.6 (3.3) cycles∙min^−1^ and 0.13 (0.09) m∙cycles^−1^; Figure 3).

## 4. Discussion

The purpose of the current study was to validate the physiological and biomechanical parameters corresponding to a fixed lactate concentration of 4 mmol∙L^−1^ with those measured in an intermittent 5 × 400-m swimming training set at constant speed. A constant swimming speed corresponding to V4 was successfully maintained and BL was no different from that at 4 mmol∙L^−1^ during the 5 × 400-m training set. However, during the training set, a lower SL and a higher HR and SR compared to predicted values were recorded. Despite the Bland and Altman plot indicating agreement, a great individual variation was observed for all physiological and biomechanical variables.

A similar speed compared to V4 was expected during the 5 × 400-m training set as this was predesigned in our experimental design. This is because swimmers were guided to follow this speed using audio signals. Considering that swimmers maintained the prescribed speed, we should explain the observed metabolic and biomechanical alterations required by the swimmers to maintain this speed. No difference was found between 4 mmol∙L^−1^ and BL-5×400-m, although a large effect size (*d* = 0.75) and a great variation in measured values was observed between swimmers. Using a fixed value of 4 mmol·L^−1^ has been criticized in previous studies since it does not taking into account an individual approach to estimate BL [3,12]. Specifically, it was proposed that the fixed value did not take into account the individual blood lactate concentration curve kinetics and can be affected by the muscle glycogen content [12,13]. This is confirmed by studies reporting a higher V4 or BL-V4 during a continuous swimming trial but not in an intermittent swimming set as in the current study [19,20]. However, it should be considered that in the aforementioned studies [19,20], well-trained long-distance swimmers or young swimmers were included. Well-trained swimmers specializing in various distances participated in the current study, and they may present different individual characteristics compared to swimmers participating in previous studies. The swimming stroke specialty or athlete specializations may differentiate BL efflux under a constant speed [20,21]. Specifically, it has been shown that sprint-oriented athletes or those with limited aerobic potential may present higher BL values compared with long distance athletes during a continuous swimming training set under a constant speed at V4 [21,22], as was observed in the current study. Despite the observed variation, BL concentration prescribed by the progressively increasing speed test was similar to that measured during the intermittent training set. Lactate responses are reflected by RPE, an index of internal training load, during intermittent swimming [23]; RPE calculated by the 5 × 200-m test was found to be similar to that measured during the 5 × 400-m training set.

Individual variation observed in measured BL and RPE, as well as differences in HR, may be attributed to equations used for calculation of these parameters. The second-order polynomial function used to calculate V4 presented a low error of estimation (r = 0.98, SE = 0.07) and a high accuracy of the predicted BL value. In contrast with BL, the measured HR values were higher during the 5 × 400-m intermittent training set compared to the value calculated by the 5 × 200-m progressively increasing speed test. This may be explained by a higher sympathetic activation during the 5 × 400-m intermittent training set [24]. Furthermore, the higher HR during the 5 × 400-m training set may indicate a higher effort of swimmers to maintain the prescribed V4 swimming speed. It should be noted that HR-V4 was calculated using an appropriate linear fit of data (r = 0.98) and low standard error of estimate (3 b∙min^−1^) that may partly explain the differences during 5 × 400-m training set and HR-V4. Possibly, differences in HR were dependent on exercise duration between progressively increasing speed testing protocol (200-m repetitions) and training set (400-m repetitions; [5]). In this case, HR may have not reached steady values within the shorter 200-m distance, thus underestimating the values during a longer duration 400-m distance.

Besides the physiological load required in maintaining constant V4, some differences in biomechanical parameters were also noticed. Increased SR-V4 (large effect size) and decreased SL-V4 (medium effect size) were observed during the 5 × 400-m intermittent training set compared to the calculated values by the progressively increasing speed 5 × 200-m test. Increased SR and decreased SL during swimming are connected to increased energy cost [25]. These changes occur when swimmers manage to maintain the prescribed speed. Possibly, well-trained swimmers who participated in the current study applied less force (decreased SL) to maintain an efficient arm stroke [26]. However, these changes were not severe enough to induce fatigue manifested as an inability to maintain constant speed during the 5 × 400-m. A further explanation for these differences may be that swimmers completed a higher number of arm-stroke cycles in the longer distance of 400-m compared to 200-m, thus altering their mechanics to compensate for the longer distance [27]. Possibly, the different distances used for testing (400-m vs. 200-m) as well as the swimmer’s specialty combined with the large interindividual variation between swimmers may have led to the aforementioned differences in biomechanical parameters.

The characteristics of the progressively increasing speed testing protocols (number of repetitions, duration of each repetition) that have been used to estimate physiological and biomechanical variables may lead to different responses during an intermittent or a continuous training set [28,29]. However, this is controversial throughout the literature. Specifically, Madsen et al. [30], found that a progressively increasing speed test consisted of 200-m repetitions overestimates the physiological variables obtained during continuous swimming training, while recently it has been found that 200-m and 300-m testing protocols showed similar physiological and biomechanical variables during intermittent swimming training [31]. Whatever the case, coaches should be aware that the predicted parameters are dependent on testing protocol used for their calculation and may not be similar to those expected during an intermittent training set.

## 5. Conclusions

A 5 × 200-m progressively increasing speed test and the determination of V4 calculated by interpolation of a second-order polynomial function fitted to the swimming speed vs. lactate concentration data seem to provide physiological and biomechanical variables with a large interindividual variability. It should be expected that swimmers will adjust their mechanics at increased metabolic cost to maintain the required speed during an intermittent training set including 400-m repetitions. Specifically, increased SR and decreased SL may be observed when swimmers aim to maintain speed during a 5 × 400-m intermittent training set. It seems that swimmers change the arm stroke profile in aiming to maintain the required speed during a long-duration constant intensity set. The large interindividual variation between swimmers, possibly because of their specialty or the characteristics (i.e., number of repetitions, interval time, distance) of the test used to calculate the required parameters, should be considered. Coaches should be aware that prescribed physiological or mechanical parameters may be altered when swimmers follow a training pace corresponding to V4 during aerobic endurance training.

## Figures and Tables

**Table 1:** Participant characteristics in the current study. The data are presented as mean values with SD in parentheses.

Variables	Swimmers (*n* = 12)
Age (y)	19.0 (2.2)
Body mass (kg)	74.4 (10.1)
Height (cm)	178.1 (7.9)
Fat mass (%)	13.0 (2.6)
Body Mass Index (%)	23.4 (1.5)
VO_2_peak (mL·kg^−1^·min^−1^)	65.5 (11.4)
VO_2_max (mL·kg^−1^·min^−1^)	51.2 (14.1)
Time (s), 200-m front crawl	127.1 (9.6)
FINA points, 200-m front crawl	534 (127)
Time (s), 400-m front crawl	288.7 (22.0)
FINA points, 400-m front crawl	501 (186)
FINA points of best competition	625 (149)
